# Fluorescent *In Situ* Folding Control for Rapid Optimization of Cell-Free Membrane Protein Synthesis

**DOI:** 10.1371/journal.pone.0042186

**Published:** 2012-07-27

**Authors:** Annika Müller-Lucks, Sinja Bock, Binghua Wu, Eric Beitz

**Affiliations:** Pharmaceutical and Medicinal Chemistry, Christian-Albrechts-Universität zu Kiel, Kiel, Germany; University of Oulu, Finland

## Abstract

Cell-free synthesis is an open and powerful tool for high-yield protein production in small reaction volumes predestined for high-throughput structural and functional analysis. Membrane proteins require addition of detergents for solubilization, liposomes, or nanodiscs. Hence, the number of parameters to be tested is significantly higher than with soluble proteins. Optimization is commonly done with respect to protein yield, yet without knowledge of the protein folding status. This approach contains a large inherent risk of ending up with non-functional protein. We show that fluorophore formation in C-terminal fusions with green fluorescent protein (GFP) indicates the folding state of a membrane protein in situ, i.e. within the cell-free reaction mixture, as confirmed by circular dichroism (CD), proteoliposome reconstitution and functional assays. Quantification of protein yield and in-gel fluorescence intensity imply suitability of the method for membrane proteins of bacterial, protozoan, plant, and mammalian origin, representing vacuolar and plasma membrane localization, as well as intra- and extracellular positioning of the C-terminus. We conclude that GFP-fusions provide an extension to cell-free protein synthesis systems eliminating the need for experimental folding control and, thus, enabling rapid optimization towards membrane protein quality.

## Introduction

The cloning of the GFP gene [1] has initiated a multitude of applications. One is its use as a “folding indicator”, which has been intended to evolve proteins by mutation for improved expression in *Escherichia coli* [2]. To this end, constructs are generated of the test protein with GFP fused to its C-terminus and formation of the GFP fluorophore is monitored as a measure of the amount of correctly folded protein, see Drew *et al.* for protocols [3]. Besides expression in *E. coli*, the technique is compatible with *in-vitro* translation [2], and with expression using yeast [4] and insect cells [5]. A cell-free system [6], [7] is particularly attractive for the production of membrane proteins for several reasons: high-yield at low toxicity for the setup, immediate solubilization by detergents [8], liposomes [9], or nanodiscs [10] in the reaction mixture, direct accessibility of the reaction for incorporation of isotope-labeled and unnatural amino acids, or small reaction chambers for parallelization and automation. Unfortunately, optimization of the reaction protocol, e.g. selection of a suitable detergent for protein solubilization, is limited by lengthy procedures of protein purification and structural or functional analysis to assay for correct protein folding. For this reason, cell-free synthesis conditions are usually optimized with the focus on protein yield, yet without knowledge of the folding status.

We reasoned that a GFP-fusion approach would allow us to evaluate fluorometrically the efficiency of cell-free synthesis in terms of yield of correctly folded and solubilized membrane protein directly in the reaction mixture. We chose the thoroughly characterized [11], [12] and crystallized [13] aquaglyceroporin from the malaria parasite *Plasmodium falciparum*, PfAQP, as a first six transmembrane domain test protein and generated a construct carrying a C-terminal GFP extended by a His_10_-tag. Fluorescence measurements, as well as structural and functional analyses confirmed the validity of our approach. We further show general applicability using a representative set of membrane proteins.

## Results

**Figure 1:**
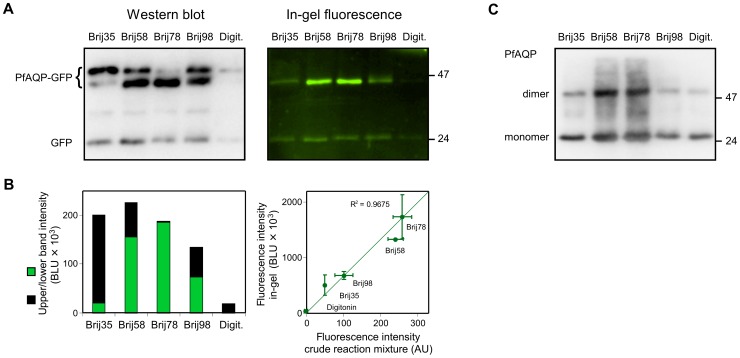
Cell-free synthesis of the *Plasmodium falciparum* aquaglyceroporin, PfAQP, with a C-terminal GFP shows detergent-dependent signal patterns. **A**. The left panel displays a Western blot of PfAQP-GFP produced in the presence of various non-ionic detergents of the Brij family and digitonin (Digit.). A polyclonal anti-GFP antiserum and a secondary horseradish peroxidase-labeled antibody were used for detection. Luminol chemiluminescence was monitored using a CCD-camera. PfAQP-GFP was obtained as two folding species with apparent molecular weights of 45 and 48 kDa. A signal representing the 24 kDa GFP domain alone was also visible. In the right panel, excitation of in-gel GFP fluorescence yielded emission signals of the 45 kDa PfAQP-GFP protein species and the 24 kDa GFP-band. **B**. PfAQP-GFP band intensities were determined semi-quantitatively by integration from the Western blot using arbitrary units (BLU – biomedical light units). The height of the bars in the left panel represents the total protein yield as a sum of the signals from the upper 48 kDa band (black) and the lower 45 kDa band (green). The plot in the right panel confirms correlation of the in-gel GFP fluorescence signal with GFP fluorometry of the crude cell-free reaction mixture. Intensity units given by the fluorometer are arbitrary (AU). The symbols and error bars denote mean values and the data range from two independent synthesis reactions and fluorometric evaluations. **C**. Cell-free synthesis, SDS-PAGE, and Western blot of non-fused PfAQP yielded signals for the monomer (≈25 kDa) and the dimer using an anti-His_5_ antiserum. A semi-quantitative representation of the protein yield is shown in Fig. S3. Most stable dimers were obtained with Brij58 and Brij78, which also led to highest in-gel fluorescence intensity.

Synthesis of PfAQP-GFP in an *E. coli* S30 extract-based cell-free system [7] using a panel of five typical non-ionic detergents for membrane protein solubilization [8] produced two bands with apparent molecular weights of 45 and 48 kDa as detected by an anti-GFP antiserum (Fig. 1A, left panel; Fig. S1). In the absence of detergent, however, we did not obtain a protein product. The total protein yield and the ratio of the upper to lower band intensity depended on the detergent (Fig. 1B, left panel), with Brij35 strongly promoting production of the upper 48 kDa band and Brij78 of the lower 45 kDa band. It has been shown before that non-monodisperse GFP-fusion proteins can differ in their SDS-PAGE mobility with the lower band representing the correctly folded, probably more compact protein [14]. When we excited GFP-fluorescence in the polyacrylamide gel (Fig. 1A, right panel) only the lower band responded with emission intensities proportional to those of the Western blot. The in-gel fluorescence signal further correlated well with fluorometry of the crude cell-free reaction mixture (Fig. 1B, right panel) allowing for in situ assessment of the fluorescent protein yield. A weak additional band of 24 kDa in both, the Western blot and the in-gel fluorescence image, corresponds in size to the GFP domain alone probably due to residual translation at the GFP start-methionine, which was present in the expression construct, or to proteolysis of the fusion protein. To test whether the presence of the in-frame ATG codon of the GFP domain was used as a translation start we generated the ATG-less version PfAQP-GFPΔATG. In gel fluorescence quantification still showed a GFP signal, yet it was reduced in intensity by a factor of three and was only visible after elongated exposure times (Fig. S2). The overexposure further revealed some weak dimerisation and probably also oligomerisation of PfAQP-GFP.

Next, we produced wild-type PfAQP without a GFP fusion under the same cell-free reaction conditions (Fig. 1C). The PfAQP protein yield obtained with each detergent is similar to that obtained with the PfAQP-GFP fusion protein (Fig. S3). Further, in vivo, aquaporins form stable homotetramers, which partially resist disintegration even during detergent treatment and often display the dimer or higher order oligomers in an SDS gel [15]. It is striking that the most stable dimers, which likely indicate correctly folded proteins, appeared in the samples with Brij58 and Brij78, i.e. the detergents that yielded the highest fluorescence intensities with PfAQP-GFP. The dimer-to-monomer intensity ratios were 1.2∶1 (Brij58 and Brij78), and 0.3–0.5∶1 (remaining detergents). Hence, the reaction conditions found by using GFP fusions appear to be equally applicable to the non-fused membrane protein. This notion is reasonable because the interaction between the detergents and the protein can be expected to occur in the membrane protein domain rather than the GFP.

**Figure 2:**
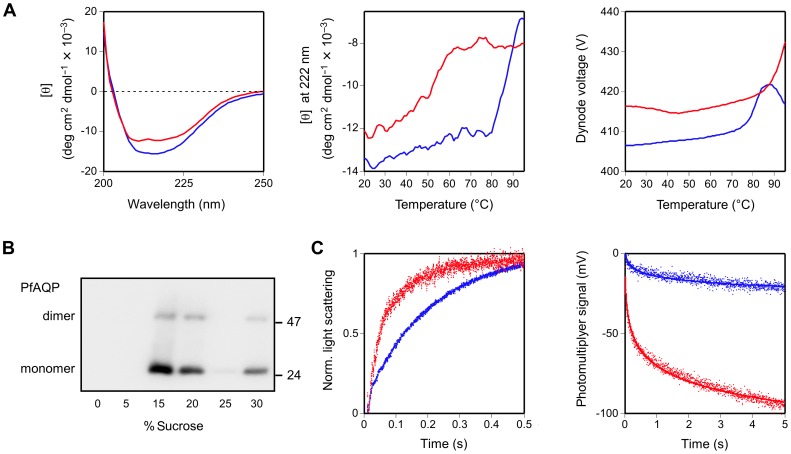
Structural and functional analysis of cell-free produced PfAQP without a GFP fusion. **A**. PfAQP in the presence of Brij35 (blue curve) and Brij78 (red curve) was analyzed by circular dichroism. The left panel shows the mean residue molar ellipticity [θ] in the range of 200–250 nm. Thermal unfolding was monitored at 222 nm from 20°C to 95°C (middle panel). A plot of the photomultiplyer dynode voltage versus temperature (right panel) indicates an increase in turbidity at 80°C in the sample with Brij35 solubilized PfAQP suggesting protein agglomeration. **B**. Reconstitution of PfAQP, produced in the prescence of Brij78, into proteoliposomes was controlled by sucrose density gradient centrifugation and Western blot using an anti-His_5_ antiserum. The fractions with 15% and 20% sucrose contained reconstituted liposomal PfAQP; the 30% sucrose fraction displays precipitated, non-integrated PfAQP protein. PfAQP monomers (≈25 kDa) and dimers are visible. **C**. For functional analysis, PfAQP-proteoliposomes (red traces) and empty control liposomes (blue traces) were subjected to an outward osmotic gradient of 300 mosm kg^−1^ (left panel) and an inward isotonic glycerol gradient of 300 mM (right panel). Changes in the light scattering intensity reflect liposome shrinkage due to water efflux (increase in light scattering) and liposome swelling due to glycerol plus secondary water influx (decrease in light scattering). Note the difference of the scale of the abscissae as a consequence of lower glycerol permeability by at least one order of magnitude. The slow glycerol flux across the plain lipid liposome membrane did not reach a plateau, hence, the photomultiplyer signal was plotted without normalization. For each experiment nine traces were averaged and fitted to single exponential functions.

We then took advantage of our finding that the choice of Brij35 resulted in mainly one PfAQP species (90% of the total protein in the upper band) whereas Brij78 almost exclusively yielded the other (98% in the lower band; see Fig. 1) and analyzed the structural features of both folding species. For the following experiments, we used affinity purified, non-fusion versions of PfAQP in order to exclude an influence of the GFP domain. At all times, the samples were kept in buffers supplemented with Brij35 or Brij78, respectively. The obtained CD spectra differed markedly in extent in the negative range (Fig. 2A, left panel). Further, deviations in shape were visible between 210 and 220 nm. The spectrum of the Brij78 solubilized PfAQP depicts a doublet of minima (red curve) clearly indicating an alpha-helical protein, which is consistent with the PfAQP crystal structure containing 74% alpha-helical structures and fully lacking beta-sheets [13]. The Brij35 solubilized PfAQP protein (blue curve), however, resulted in a wide, singlet minimum being indicative of a considerable fraction of non-native beta-sheets in the protein [16]. Determination of the melting temperatures gave an even stronger hint at structural differences between both PfAQP folding species (Fig. 2A, middle panel). PfAQP produced in Brij78 yielded an apparent melting temperature of 53°C. This is close to the value of 55°C recently found with the non-glycosylated form of a related human aquaglyceroporin, hAQP10 [17]. The much higher apparent melting temperature of approximately 80°C obtained with PfAQP in Brij35 very likely does not indicate an unfolding process of the protein because it is untypically high. It is, however, consistent with the agglomeration of denatured or misfolded proteins [18]. Protein agglomeration increases the turbidity of the solution and results in lower detector signals that can be monitored via the applied dynode voltage of the photomultiplyer. With PfAQP in Brij35, the rise of the apparent melting temperature and the dynode voltage coincide at 80°C. The drop in the dynode voltage at temperatures above 90°C may be explained by protein precipitation clearing the sample (Fig. 2A, right panel) [18].

To confirm functionality of the Brij78-solubilized PfAQP we prepared proteoliposomes (Fig. 2B). The proteoliposomes were subjected to outward-directed osmotic (Fig. 2C, left panel) and inward-directed isosmolar glycerol gradients (Fig. 2C, right panel) for assessment of water and glycerol permeability [19]. Water efflux results in liposome shrinking and an increase in light scattering [19], [29], [30]; glycerol influx leads to liposome swelling and a decrease in light scattering [19]. In both experiments, the PfAQP-containing proteoliposomes exhibited 4–5 times higher permeability rates (red traces) than pure lipid control liposomes (blue traces) indicating a functional protein.

Having established a clear correlation between fluorescence intensity of a PfAQP-GFP fusion and structural as well as functional integrity, we selected a set of representative membrane proteins to evaluate a more general applicability of GFP as a folding indicator in cell-free protein synthesis. First, we chose two equilibrative nucleoside transporters from *Arabidopsis thaliana* [20], [21], AtENT1 and AtENT3. *In vivo*, AtENT1 localizes to intracellular vacuoles [22], whereas AtENT3 resides in the plasma membrane [21]. The nucleoside transporters have eleven transmembrane spanning domains with the C-terminus positioned extracellularly [23]. The latter feature of this group of membrane proteins is of special interest for our cell-free approach, because in cell-based systems extracellular GFP usually does not form a functional fluorophore when exposed to the culture media [5]. As a test protein of mammalian origin, we selected the rat urea transporter type B, UT-B [24]. Urea transporters span the membrane ten times and additionally carry two pore helices [25]. Finally, we used the *E. coli* formate transporter, EcFocA, with six transmembrane domains [26]. EcFocA does not show any sequence similarity with aquaporins but shares the same fold.

**Figure 3:**
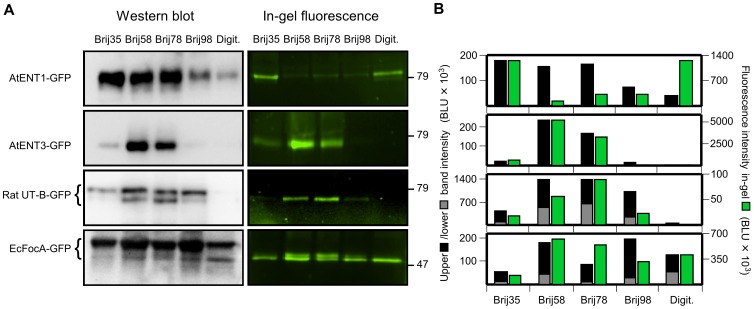
Semi-quantification of protein yield and in-gel GFP fluorescence of representative membrane protein-GFP fusions. **A**. Shown are Western blot (left panel) and in-gel fluorescence signals (right panel) of GFP fusions with *Arabidopsis* nucleoside transporters, AtENT1 and AtENT3, the rat urea transporter B, UT-B, and the *E. coli* formate transporter, EcFocA, after cell-free synthesis in the presence of various detergents of the Brij-type and digitonin (Digit.). For membrane protein characteristics see text. The molecular weight of marker proteins is indicated on the right. **B**. The black and gray bars represent intensities of the upper and lower band from the Western blot and the green bars those of the in-gel GFP fluorescence. The intensity units are arbitrary (BLU – biomedical light units). The differences in scale of the ordinates derive from different exposure times used in the individual experiments.

Quite expectedly, we obtained individual patterns of detergent-dependent protein yield as determined by Western blot and in-gel GFP fluorescence intensity with each protein (Fig. 3A). It is noteworthy that total protein yield was not a predictor of fluorescence intensity (Fig. 3B). In fact, a direct correlation between the amount of total protein and fluorescence intensity was found only for AtENT3-GFP suggesting that in this case all produced protein was correctly folded. Cell-free synthesis of rat UT-B-GFP and EcFocA-GFP yielded polydisperse protein products. As with PfAQP-GFP the lower bands corresponded to the fluorescent, presumably correctly folded species. EcFocA synthesis further produced a double fluorescent band with Brij58, Brij78, and Brij98, which may hint at an additional folding species that was compatible with the formation of the GFP fluorophore. The example of AtENT1 underscores the value of the GFP-fusion approach particularly well. Optimization of the reaction conditions towards maximal protein yield would have led to the use of either Brij35, Brij58, or Brij78 and digitonin would have been omitted (Fig. 3A, left panel). The Western blot gives no indication of more than one folding species. However, in-gel GFP fluorescence quantification revealed that at least 80% of the Brij35-solubilized protein was misfolded as seen by equal fluorescence intensity in the digitonin sample, which yielded only 20% of the total protein (Fig. 3B). With Brij58 or Brij78 this discrepancy would have been much higher and the chance of obtaining functional protein probably minuscule.

## Discussion

Cell-free production typically yields considerable amounts of protein in a short period of time. Due to the small volume of the synthesis chambers multiple reactions can be set up in parallel. A good indicator for the quality of soluble proteins is the quantity ratio of precipitated, i.e. most likely misfolded, protein to the protein in the supernatant, possessing a high likelihood of being correctly folded. Controlling the folding of membrane proteins is much more time consuming and requires structural and/or functional analyses of the purified and solubilized or reconstituted membrane protein. Hence, there is a large discrepancy in hands-on time between membrane protein production and quality control, which prevents automated high-throughput optimization of the reaction conditions. This problem of time and cost demanding quality control is evident even with smaller sets of membrane proteins often leading to optimization of the cell-free reaction protocol simply towards maximal protein yield. Data from our test set of membrane proteins indicates that this is the wrong way to go and, more often than not, one will end up with high amounts of protein, yet of bad quality. To circumvent this major shortcoming and putative source of frustration an immediate and simple to read out indicator is urgently needed.

From our data it is evident that the selection of a detergent influences both, total protein yield and obtained GFP fluorescence intensity, independently. The detergents of our set were compatible with *E. coli* based cell-free protein synthesis, i.e. they did not affect the ribosomal translation process in general. Yet, certain combinations of detergent and membrane protein to be produced resulted in a block of synthesis, e.g. digitonin with AtENT3-GFP and rat UT-B-GFP, while the same detergent facilitated production of other membrane proteins, e.g. digitonin with AtENT1-GFP and EcFocA-GFP (Fig. 3A). We propose that solubilization by a detergent already of the nascent protein chain is required to maintain the ribosomal translation process. If this fails protein production will likely cease. This notion is further supported by the lack of PfAQP protein product in the absence of a detergent and observations by others [7], [8]. We can now show that solubilization alone does not necessarily lead to correct folding as indicated by the ratios of total protein to GFP-fluorescence (Figs. 1B and 3B). Furthermore, the non-native beta-sheet content of the misfolded PfAQP species in Brij35 fits well into this scenario. Chow *et al.* [16] found that nascent alpha-helical proteins can undergo a conversion from initially present beta-sheets to alpha-helices with increasing peptide chain length. The respective CD spectra documenting this process depict a change in shape from a singlet to a doublet minimum in the 210–220 nm range, which is highly reminiscent of the spectra obtained with PfAQP in Brij35 vs. Brij78 (Fig. 2A, left panel). Finally, since the formation of the GFP fluorophore occurs in the minute range [27] our approach can be used to monitor the folding process within the crude cell-free reaction mixture, i.e. in situ, and provides options for automation in high-throughput applications.

Together, our study shows that GFP is exploitable as a folding indicator in cell-free membrane protein synthesis using an *E. coli* S30 extract. Transfer to eukaryotic cell-free systems [6] should be straightforward. In fact, recently, fusion constructs of several membrane proteins with an N-terminal GFP were produced in a cell-free wheat germ based system using the GFP for visualization of the protein integration into liposomes. Usage of a C-terminal GFP instead probably could have served as an additional folding control [28]. The GFP domain can be subsequently removed from the fusion protein by proteolysis during protein purification, or the optimized reaction conditions can be applied to the non-fused membrane protein for large-scale production. Fluorescence indication can be done in the crude reaction mixture, requires only small sample volumes, and can be automated. We conclude that the use of membrane protein-GFP fusions eliminates the bottleneck of experimental analysis of the protein fold leading to rapid cell-free optimization results.

## Materials and Methods

### Cloning of the GFP-fusion constructs

The pIVEX2.3 plasmid (Roche Diagnostics GmbH, Mannheim, Germany) was modified by digestion at its Nco I and Bpu1102 I restriction sites and subsequent ligation with synthetic oligonucleotides to extent the multiple cloning site and to encode an N-terminal hemagglutinin-tag (YPYDVPDYA) and a C-terminal His_10_-tag yielding pIVEX23w. For synthesis of proteins with a C-terminal GFP fusion, mGFP5 [29] including an N-terminal linker sequence (RPACKIPNDLKQKV) was amplified by PCR from the pmGFPc plasmid (Lab collections) using primers that introduce flanking Xho I and Sal I restrition sites. The PCR product was ligated into the Xho I site of pIVEX23w to obtain pIVEX23wGFP. Both plasmid sequences were confirmed by DNA sequencing. The plasmids PfAQP-pIVEX23w and PfAQP-pIVEX23wGFP were constructed by PCR amplification of the 774 bp coding sequence of PfAQP (NCBI GeneID 810885) [11] without the stop codon and ligation of the PCR product into the BamH I and Xho I sites of both pIVEX23w and pIVEX23wGFP. DNA coding for EcFocA (NCBI GeneID 945513) [26] (855 bp without the stop codon) was amplified from *E. coli* DH5-alpha genomic DNA and ligated into the BamH I and Xho I sites of pIVEX23w and pIVEX23wGFP. cDNA encoding the rat urea transporter UT-B (NCBI GeneID 54301) [24] was kindly provided by O. Fröhlich (Emory University, Atlanta, Georgia). The coding sequence without the stop codon (1167 bp) was ligated into the BamH I and Hind III sites of pIVEX23w and pIVEX23wGFP. cDNAs encoding the *Arabidopsis thaliana* equilibrative nucleoside transporters [20], [21] AtENT1 and AtENT3 were a gift from T. Möhlmann (University of Kaiserslautern, Germany). The coding sequences of AtENT1 (1350 bp; NCBI GeneID 843369) and of AtENT3 (1254 bp; NCBI GeneID 825857), both without stop codons, were cloned into the BamH I and Xho I sites of both pIVEX23w and pIVEX23wGFP. The PfAQP-GFPΔATG construct was generated by site-directed mutagenesis using the QuikChange protocol (Stratagene). All constructs were subjected to DNA sequencing for verification.

### Cell-free protein synthesis

Proteins were synthesized in an *E. coli* S30 extract-based continuous exchange cell-free (CECF) setup with coupled transcription/translation and T7 RNA polymerase control according to prior protocols [7], [30]. The S30 extract was prepared from *E. coli* strain BL21. For analytical scale syntheses, reaction chambers were covered with a 14 kDa cut-off cellulose membrane and filled with 55 µl of reaction mix containing 1 µg of the respective expression plasmid and a detergent. Final detergent concentrations [8] were: Brij35 (0.2%), Brij58 (1.5%), Brij78 (0.8%), Brij98 (0.2%) and digitonin (0.4%). The chambers were placed in 850 µl of feeding mix solution, i.e. a reaction-to-feeding mix volume ratio of 1∶15. For preparative scale, 0.5–3 ml of reaction mix were filled into a Slide-A-Lyzer dialysis cassette (Pierce) and placed into a feeding mix bath with a volume ratio of 1∶17. Incubation was done overnight at 30°C in a shaking water bath. The samples were subsequently stored on ice.

### Protein Purification

For analytical scale, 400 µl of purification buffer (300 mM NaCl, 0.05% n-dodecyl-ß-maltoside, 20 mM Tris-HCl, pH 8.0) and 15 µl of prewashed Ni-NTA agarose slurry (Qiagen) were added to 55 µl of crude reaction mix and shaken for 3 h at room temperature. The slurry was washed two times with 75 µl purification buffer supplemented with 20 mM imidazole. The protein was eluted in 80 µl purification buffer supplemented with 300 mM imidazole. For preparative scale, 4 ml of purification buffer and 200 µl of prewashed Ni-NTA slurry were added per 1 ml of crude reaction mix and shaken overnight at 4°C. Washing and elution was done stepwise in fractions of 500 µl by using purification buffer supplemented with increasing imidazole concentrations from 20 mM to 300 mM. Imidazole was removed and protein was concentrated using centrifugal filter devices (Amicon Ultra-4 10 k, Millipore).

### In-gel fluorescence monitoring and Western blot analysis

10 µl of the purified protein solutions were heated to 37°C for 30 min in SDS-loading buffer and electrophoretically separated using 15% SDS polyacrylamide gels [14]. In-gel GFP fluorescence is stable even under denaturing conditions due to the covalent nature of the fluorophore and was monitored at 520 nm using the Lumi-Imager F1 (Roche) and evaluated using the LumiAnalyst 3.1 software (Roche). For subsequent Western blotting, the proteins were transferred to PVDF membranes and probed with a rabbit polyclonal anti-GFP antiserum (Santa Cruz Biotechnology), a mouse monoclonal anti-hemagglutinin antibody (Roche), or a mouse monoclonal penta-His antibody (Qiagen). Secondary horseradish peroxidase-conjugated antibodies (Jackson Immuno Research) were used for detection with Amersham ECL plus reagents (GE Healthcare). Chemiluminescence was quantitated using the Lumi-Imager F1 (Roche).

### Fluorescence spectroscopy

200 µl per sample of crude cell-free reaction mix were placed into 96-well plates (OptiPlate-96, White Opaque, PerkinElmer) and read out using the LS55 Fluorescence Spectrometer (PerkinElmer) at 475 nm excitation and 509 nm emission wavelenghts. Samples were diluted if required.

### Preparation of proteoliposomes

Preparation was done following the protocol by Verdoucq *et al.*, 2006 [31]. Briefly, 2.5 mg ml^−1^ *E. coli* polar lipids (Avanti Polar Lipids) were dissolved in diethyl ether and subsequently evaporated in a stream of nitrogen for 1 h. The thin lipid film was resuspended in 30 mM KCl, 20 mM Tris/MES, pH 8.0 by vortexing with glass beads. Afterwards, Triton X-100 was added dropwise to a final concentration of 0.5%. The lipid solution was combined with the purified preparative scale membrane protein in a lipid-to-protein ratio of 10 and incubated for 30 min at room temperature. Proteoliposomes were formed by removing the detergent with Bio-Beads SM-2 adsorbent (Bio-Rad) at 4°C overnight, followed by another 3 h incubation with fresh Bio-Beads. Finally, the proteoliposomes were extruded at room temperature (LiposoFast, Avestin) using 0.2 µm polycarbonate filters (Nucleopore Track-Etched Membranes, Whatman). Control liposomes were prepared simultaneously without addition of the protein. Particle size homogeneity of the proteoliposomes and empty liposomes was confirmed by dynamic light scattering (Zetasizer Nano ZS, Malvern Instruments).

### Sucrose gradient centrifugation

The sucrose gradient was formed [32] by layering 700 µl each of 30%, 25%, 20%, 15%, 5%, and 0% (w/v, bottom to top) solutions of sucrose in 100 mM NaCl, 20 mM Tris, pH 7.5. The 30% sucrose layer was prepared from 350 µl of 60% sucrose buffer plus 350 µl of proteoliposome suspension (approx. 50 µg total protein). The samples were centrifuged at 164 000×g, 4°C for 6 h using an SW60 Ti swing-out rotor (Beckman Coulter). For Western blot analysis, protein from each fraction was precipitated by adding an equal volume of 10% trichloroacetic acid.

### CD spectroscopy

PfAQP synthesized in the presence of Brij35 or Brij78 was purified as described above except that the purification buffer contained 0.2% Brij35 or 0.8% Brij78 instead of n-dodecyl-ß-maltoside. CD measurements were carried out at room temperature (J-715 CD spectrometer, Jasco) using a cuvette of 1 mm path length and a protein concentration of 0.188 mg ml^−1^ (Brij35) or 0.173 mg ml^−1^ (Brij78) as determined at 280 nm (Nano Drop Spectrophotometer ND1000, Peqlab). Four scans taken at a speed of 5 nm min^−1^ were averaged and spectra taken with buffer were subtracted. Thermal unfolding in the range of 20 to 95°C was monitored at 222 nm and blank spectra were subtracted.

### Stopped-flow light scattering measurements

Proteoliposomes were diluted to a final lipid concentration of approximately 0.4 mg ml^−1^ and equilibrated in solutions of 30 mM KCl, 20 mM TRIS/MES, pH 8.0 for osmotic water permeability measurements and of 600 mM sucrose, 30 mM KCl, 20 mM TRIS/MES, pH 8.0 for isotonic glycerol permeability measurements. Measurements were carried out at 10°C using a stopped-flow apparatus (SFM-300, BioLogic) [19], [29]. For water permeability measurements, proteoliposomes were rapidly mixed with the same volume of hyperosmolar buffer (600 mM sucrose, 30 mM KCl, 20 mM TRIS/MES, pH 8.0) generating a 300 mosm kg^−1^ outward osmotic gradient. For isotonic glycerol measurements, proteoliposomes were rapidly mixed with the same volume of isotonic buffer (300 mM glycerol, 300 mM sucrose, 30 mM KCl, 20 mM TRIS/MES, pH 8.0) generating a 300 mM inward glycerol gradient. Changes in 90° light scattering were followed at 546 nm. For each measurement 9 curves were averaged and fitted to single exponential equations.

## Supporting Information

Figure S1 **Western blot of PfAQP-GFP probed with an anti-hemagglutinin antiserum directed against the N-terminal hemagglutinin epitope tag, which was present in the expression construct.** PfAQP-GFP was detected in two folding species with apparent molecular weights of 45 and 48 kDa identical to the Western blot using an anti-GFP antiserum (Fig. 1A, left panel). This shows that the Western blot signals are specific and both termini of the PfAQP-GFP protein are present. A signal representing the 24 kDa GFP domain alone was not detected since this domain does not contain the hemagglutinin epitope.
(TIF)

Figure S2 **Cell-free synthesis of an PfAQP-GFP mutant lacking the in-frame ATG start codon of the GFP-domain analyzed by in-gel GFP fluorescence imaging.** The fluorescence intensity of the 24 kDa GFP signal is reduced by a factor of three, yet, remains detectable after longer exposure times. Equally, PfAQP-GFP dimers and oligomers are visible in the lanes showing Brij58 and Brij78 solubilized protein.
(TIF)

Figure S3 **Semi-quantitative representation of the yield of non-fused PfAQP protein obtained from cell-free synthesis in the presence of various detergents of the Brij-family and digitonin.** The yield is similar to that of the PfAQP-GFP fusion protein under the same respective synthesis conditions (Fig. 1B).
(TIF)
