# Supplementary figures and images for: Fluorescent In Situ Folding Control for Rapid Optimization of Cell-Free Membrane Protein Synthesis
Source: PLoS One. 2012 Jul 27;7(7):e42186. doi: 10.1371/journal.pone.0042186 (PMC3407079; doi:10.1371/journal.pone.0042186)

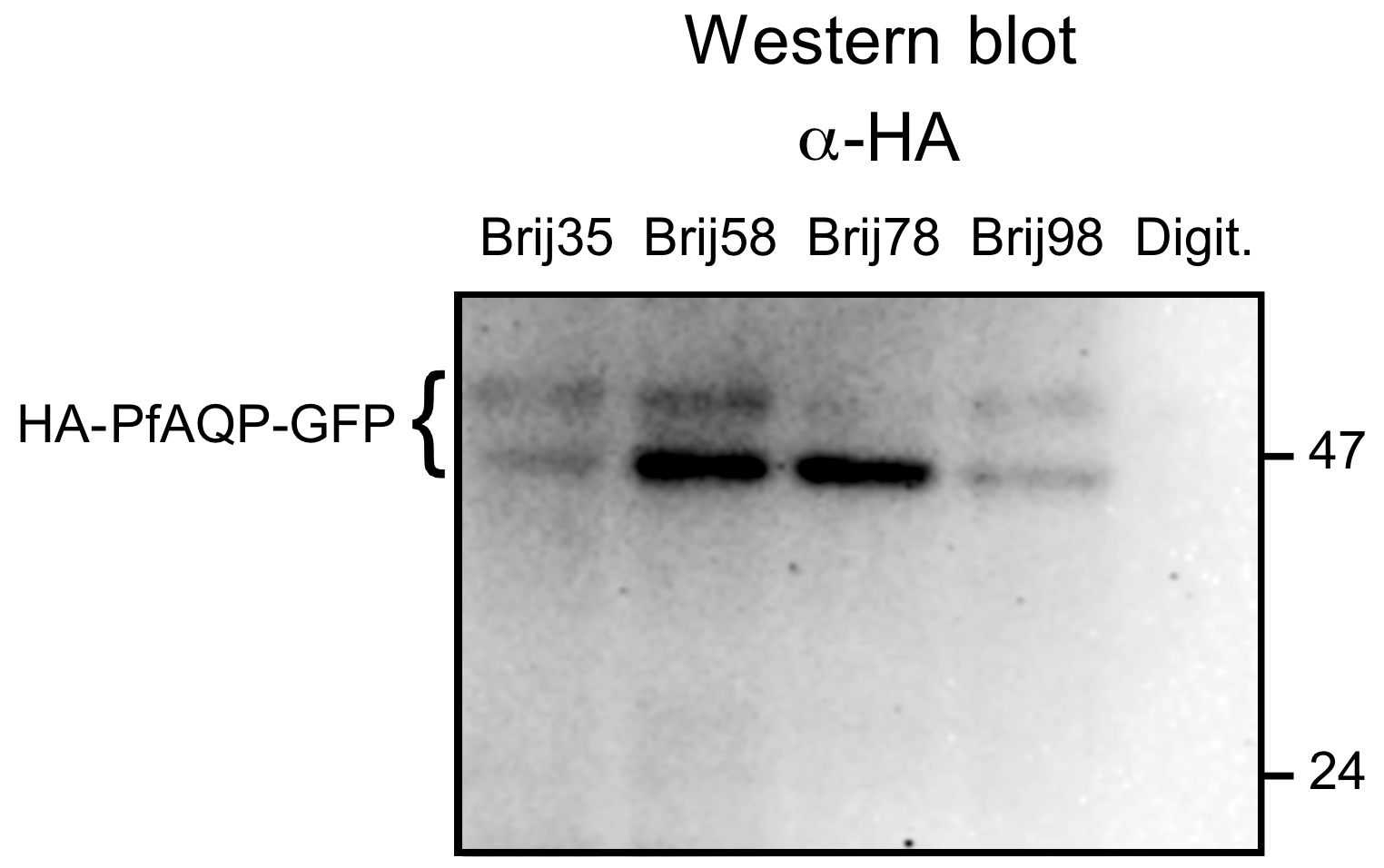

Supplement: Figure S1 — Western blot of PfAQP-GFP probed with an anti-hemagglutinin antiserum directed against the N-terminal hemagglutinin epitope tag, which was present in the expression construct. PfAQP-GFP was detected in two folding species with apparent molecular weights of 45 and 48 kDa identical to the Western blot using an anti-GFP antiserum (Fig. 1A, left panel). This shows that the Western blot signals are specific and both termini of the PfAQP-GFP protein are present. A signal representing the 24 kDa GFP domain alone was not detected since this domain does not contain the hemagglutinin epitope. (TIF) [file pone.0042186.s001.tif]

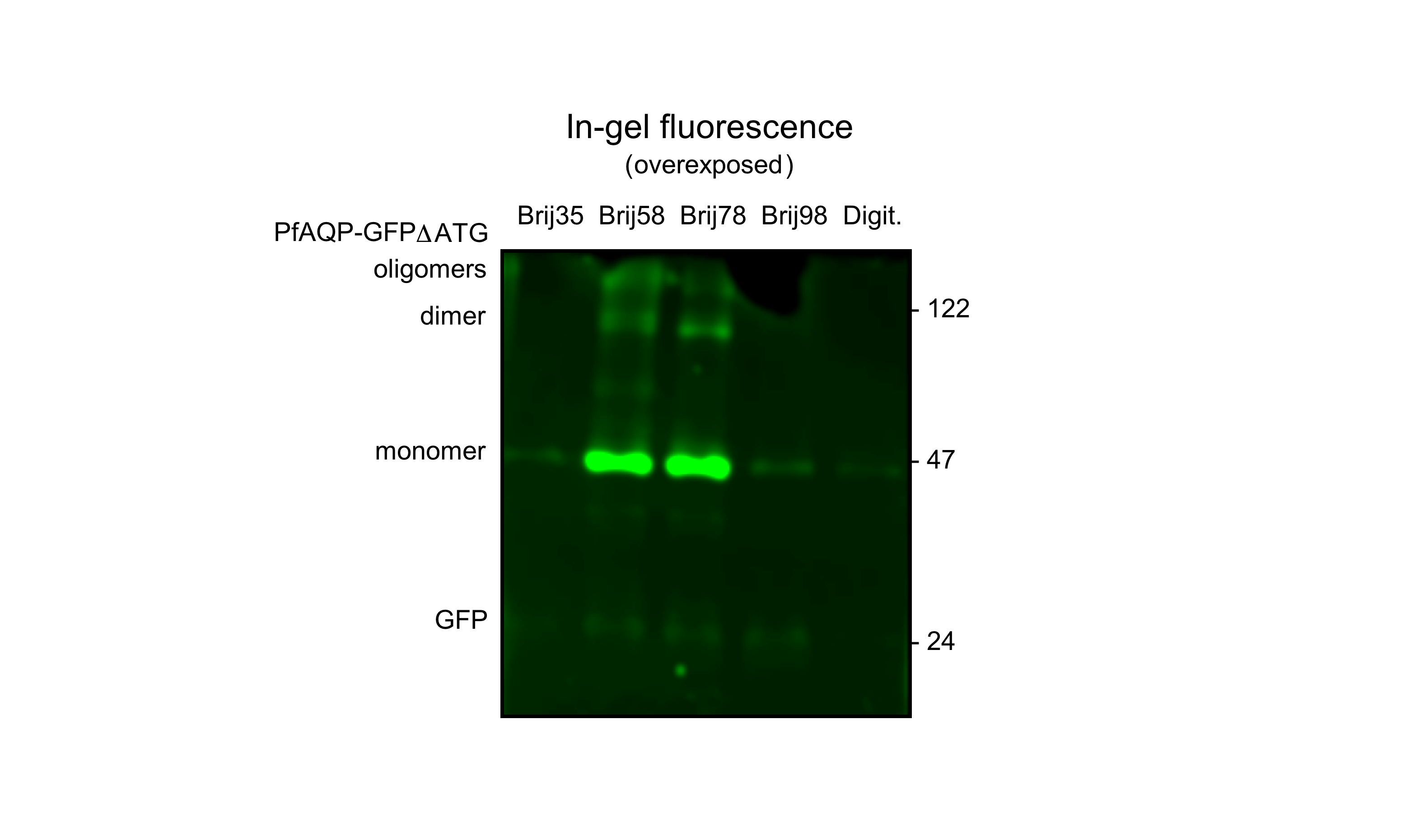

Supplement: Figure S2 — Cell-free synthesis of an PfAQP-GFP mutant lacking the in-frame ATG start codon of the GFP-domain analyzed by in-gel GFP fluorescence imaging. The fluorescence intensity of the 24 kDa GFP signal is reduced by a factor of three, yet, remains detectable after longer exposure times. Equally, PfAQP-GFP dimers and oligomers are visible in the lanes showing Brij58 and Brij78 solubilized protein. (TIF) [file pone.0042186.s002.tif]

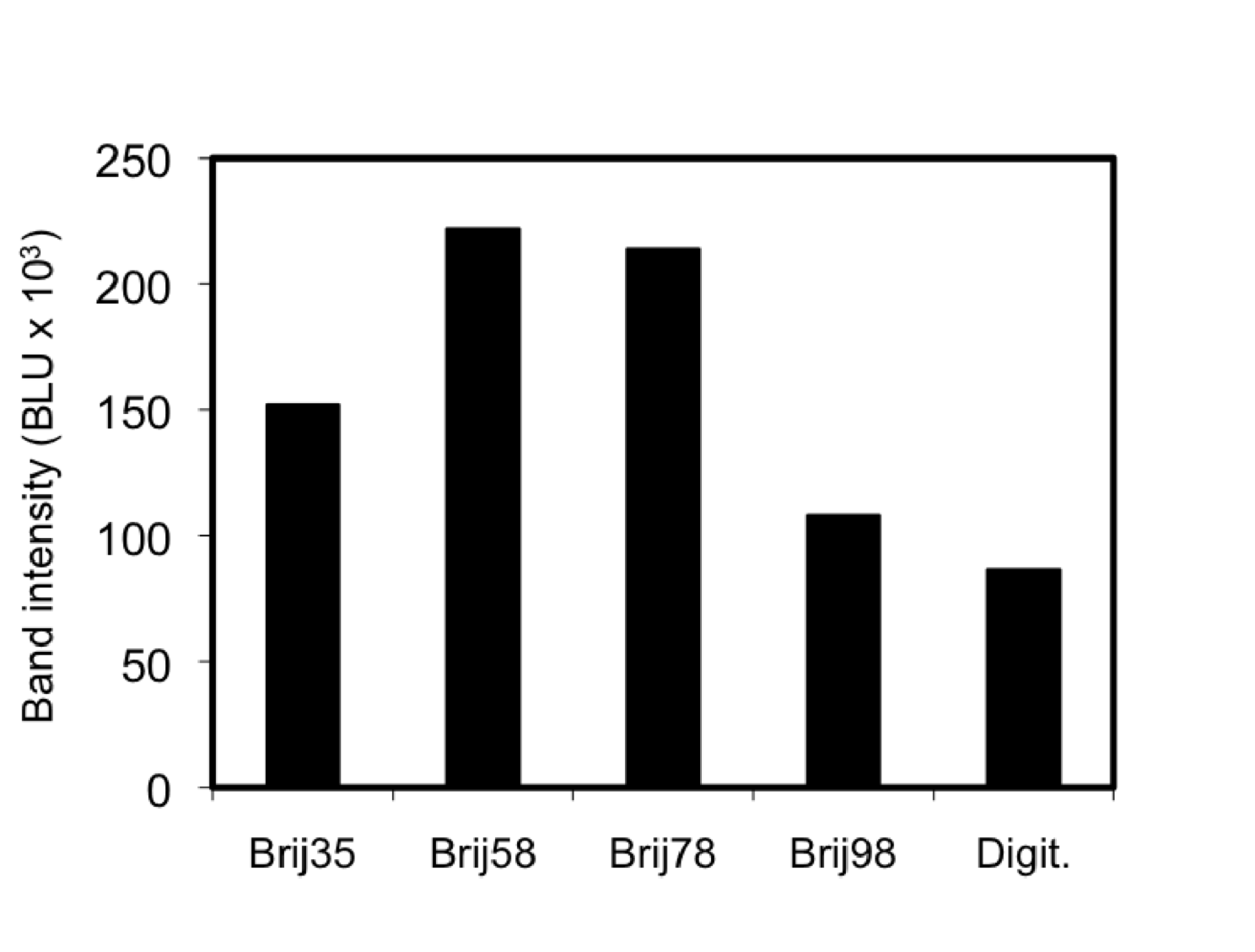

Supplement: Figure S3 — Semi-quantitative representation of the yield of non-fused PfAQP protein obtained from cell-free synthesis in the presence of various detergents of the Brij-family and digitonin. The yield is similar to that of the PfAQP-GFP fusion protein under the same respective synthesis conditions (Fig. 1B). (TIF) [file pone.0042186.s003.tif]
